# Development of an M cell targeted nanocomposite system for effective oral protein delivery: preparation, in vitro and in vivo characterization

**DOI:** 10.1186/s12951-020-00750-y

**Published:** 2021-01-09

**Authors:** Jae Geun Song, Sang Hoon Lee, Hyo-Kyung Han

**Affiliations:** BK21 FOUR Team, College of Pharmacy, Dongguk University-Seoul, Dongguk-ro-32, Ilsan-Donggu, Goyang, Korea

**Keywords:** Insulin, Oral delivery system, M cell targeting, Nano-carrier, Aminoclay

## Abstract

**Background:**

There is a strong need for non-invasive and patient-friendly delivery systems of protein drugs for long-term therapy. However, oral delivery of protein drugs is a big challenge due to many barriers including instability in the gastrointestinal (GI) tract and low permeability. To overcome the absorption barriers in GI tract and improve the patient compliance, this study aimed to develop an M cell targeted-nanocomposite delivery system of protein drugs.

**Results:**

An aminoclay-protein core complex (AC-Ins) was prepared by using insulin as a model protein and then sequentially coated with *Ulex europaeus agglutinin* 1 (UEA-1) for M-cell targeting and the pH sensitive polymer, Eudragit® L100 (EUAC-Ins). All nanoparticles were obtained with a high entrapment efficiency (> 90%) and their structural characteristics were confirmed by Fourier transform-infrared spectroscopy, energy dispersive X-ray spectroscopy, and circular dichroism. Among the developed nanoparticles, EUAC-Ins effectively suppressed drug release at pH 1.2, while rapidly released drugs at pH 6.8 due to dissolution of the outer coating layer. The conformational stability of insulin entrapped in EUAC-Ins was well maintained in the presence of proteolytic enzymes. Compared to free insulin, EUAC-Ins increased the membrane transport of insulin by 4.4-fold in M cells. In parallel, oral administration of EUAC-Ins in mice enhanced insulin uptake by 4.1-fold in the intestinal Peyer’s patches and 2.6-fold in intestinal epithelium tissues with normal villi, compared to free insulin. Orally administered EUAC-Ins decreased significantly the blood glucose level in diabetic mice, while the effect of oral insulin solution was negligible.

**Conclusion:**

An M cell targeted-ternary nanocomposite system obtained by dual coating of the aminoclay-protein core complex with UEA-1 and a pH dependent polymer is promising as an effective oral protein delivery carrier. 
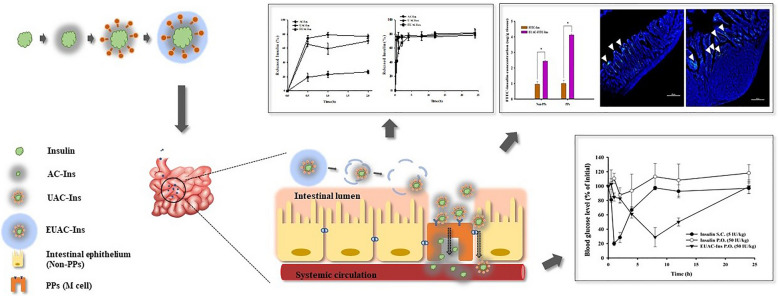

## Background

Peptides and proteins have great potential as therapeutic drugs for various incurable diseases, and their global market is rapidly growing [1, 2]. Among the many therapeutic proteins, insulin plays a key role in the treatment of diabetes. While the number of patients with diabetes has continuously increased worldwide, current treatment methods (self-injection of insulin) are unfavorable due to inherent issues of injectable formulations, such as immune reactions, infections, pain, and discomfort [3–5]. Therefore, there is a strong need for non-invasive and patient-friendly delivery systems of insulin for long-term therapy.

In general, oral administration is the preferred route due to high patient compliance. However, there are many complications in the oral delivery of proteins because of physicochemical instability, low permeability, and susceptibility to enzymatic degradation in the gastrointestinal (GI) tract [6–9]. Many formulations have attempted to overcome these obstacles, including liposomes, emulsions, and polymeric nanoparticles [10]. Microfold cell (M cell) targeted delivery systems or pH sensitive delivery systems have also been explored to improve oral delivery of proteins [11–14]. In addition to individual approaches, combined M cell targeted and pH sensitive systems might be useful to enhance GI stability and intestinal uptake of protein drugs. First, M cells are located in the follicle-associated epithelium of Peyer’s patches (PPs) that are the gatekeepers of the mucosal immune system [15, 16]. As a principal site of antigen entry, M-cells transcytose the antigen from the gut lumen to the underlying lymphoid tissues to generate a mucosal immune response [15, 16]. In addition, M cells with high transcytosis capacity and basolateral lymphocyte-containing pockets efficiently transport a wide variety of macromolecules from the gut lumen to the inside of the PPs. As M cells can translocate diverse particulates without digesting them, they have been used to develop micro-/nano-particles for various macromolecules [15, 16]. Researchers have attempted to develop M cell targeted drug carriers using lectins that bind to carbohydrate residues on the surface of M cells [7, 17]. Previous research has demonstrated the effectiveness of M cell targeting by *Ulex europaeus agglutinin* 1 (UEA-1), a representative lectin that interacts with α-l-fucose residues on the apical surface of M cells [11, 18, 19]. Second, surface coating with a pH-sensitive polymer can prevent the immature release of proteins in the harsh gastric environment and deliver more drugs to the upper and/or lower intestines. Accordingly, polymers with pH-dependent solubility have been adopted to control drug release sites during transition along the GI tract. For example, Eudragit® L-100 is soluble at intestinal pH values higher than 6.0 and is used to develop pH-dependent drug delivery systems [12, 13, 20, 21]. Therefore, dual surface coating using M cell-targeted ligand and pH-dependent polymers could enhance the oral bioavailability of proteins. In addition to surface coating, the effectiveness of core nanoparticles as drug carriers is also important to improve the oral bioavailability of proteins.

This study prepared core nanoparticles using aminoclay (3-aminopropyl functionalized magnesium phyllosilicate) as a drug carrier. Aminoclay is a synthetic organic–inorganic layered material that is delaminated to water-soluble cationic nanosheets in water. It can interact with negatively charged drug molecules to produce the drug-clay complex [22]. Previous research has demonstrated that the formation of the drug-clay complex improved the water solubility and bioavailability of drugs [23]. Aminoclay has several advantages as a drug carrier, including (i) easy preparation of a nanocomplex via spontaneous co-assembly with a diverse range of drugs, (ii) an effective support matrix to maintain the native structure of immobilized proteins, (iii) a reversible effect on tight junction opening, and (iv) low cytotoxicity [23–29]. Han et al. [28] demonstrated that aminoclay had little cytotoxicity at concentrations of up to 500 μg/mL and did not induce apoptosis at concentrations of up to 1000 μg/mL. Furthermore, Yang et al. [29] investigated the in vivo fate of aminoclay nanoparticles in mice and elucidated the low-risk of long-term tissue accumulation owing to the rapid and complete elimination of aminoclay nanoparticles via the urine and feces. As aminoclay exhibits many desirable attributes as a drug delivery carrier [22, 23, 30], we selected insulin as a model drug and prepared a nanocomplex of insulin with aminoclay (AC-Ins) by spontaneous co-assembly to develop an effective oral protein delivery system. This nanocomplex was then sequentially coated with UEA-1 for M cell targeting (UAC-Ins) and Eudragit® L-100 (EUAC-Ins), a pH-sensitive polymer. We evaluated the structural and in-vitro characteristics of the obtained nanoparticles and the in-vivo effectiveness in diabetic mice.

## Results and discussion

### Structural characterization of nanoparticles

The characteristics of the obtained nanoparticles are summarized in Table 1. All nanoparticles exhibited high entrapment efficiency (> 90%) with a narrow size distribution. First, the insulin-aminoclay nanocomplex (AC-Ins) was prepared via electrostatic interaction with an average size of 210 ± 35.5 nm and a zeta-potential of − 12.3 ± 0.50 mV. Sequential surface coating of AC-Ins with UEA-1 and Eudragit® L-100 increased the size of nanoparticles and reversed their surface charge (Table 1). As UEA-1 has an isoelectric point between pH 4.5–5.1 [31], it exhibits the positive zeta-potential (32.8 ± 0.954) in weak acidic conditions. Therefore, the zeta-potential of nanoparticles was changed to positive after surface coating with UEA-1. Similarly, the surface coating of UAC-Ins with negatively charged Eudragit® L-100 reversed the surface charge of the nanoparticles, resulting in the negative zeta-potential of EUAC-Ins (Table 1). The amount of UEA-1 coating on the nanoparticles was approximately 0.18 ± 0.01 mg/mg nanoparticles, which was determined as the difference between the lectin added initially and the lectin recovered in the solution after incubation with the nanoparticles [32, 33]. The surface coating of nanoparticles by UEA-1 was also confirmed by a confocal microscopic analysis of nanoparticles coated with FITC-labelled UEA-1 (Additional file 1: Figure S1).

**Table 1:** Characteristics of insulin-loaded nanoparticles (mean ± SD, n = 3)

Formulation	Size (nm)	PDI	Zeta potential (mV)	EE (%)
AC-Ins	210 ± 35.5	0.24 ± 0.01	− 12.3 ± 0.50	99.6 ± 0.02
UAC-Ins	236 ± 34.6	0.25 ± 0.01	38.7 ± 0.17	93.8 ± 0.31
EUAC-Ins	391 ± 42.7	0.38 ± 0.02	− 23.7 ± 1.42	94.8 ± 0.33

**Fig. 1:**
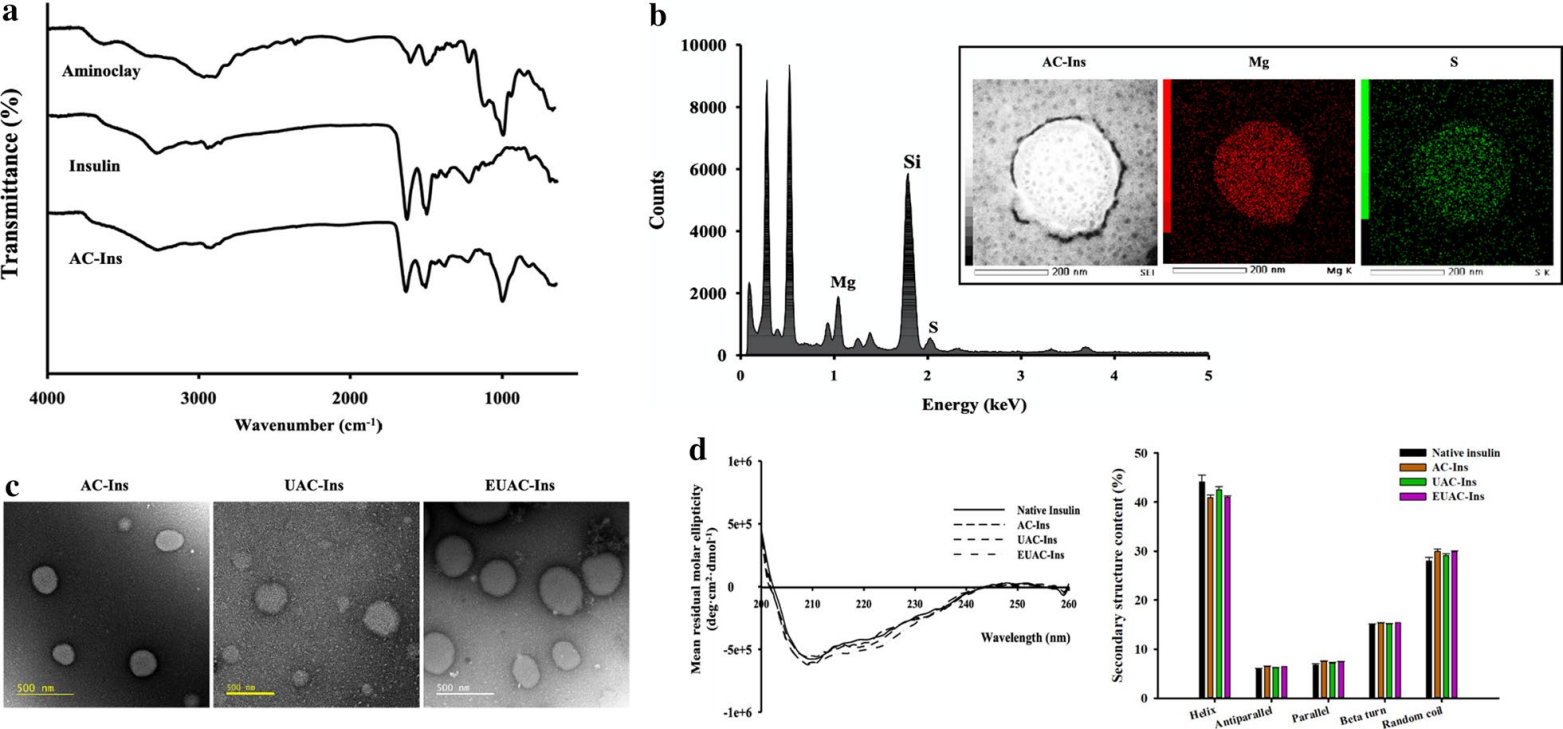
In vitro characterization of formulations. **a** FT-IR spectra of aminoclay, native insulin, and AC-Ins, **b** EDX spectrum of AC-Ins (Inset: EDX mapping analysis), **c** TEM images, and **d** CD spectra (left) and secondary structure content (right) of insulin released from each formulation after incubation at pH 6.8 for 8 h (mean ± SD, n = 3)

The formation of AC-Ins was confirmed by FT-IR and EDX analysis. As shown in Fig. 1a, the FT-IR spectrum of AC-Ins exhibited absorption bands from both insulin and aminoclay, such as peaks at 1644 cm^−1^ (amide I peak) and 1514 cm^−1^ (amide II peak) from insulin and at 1009 cm^−1^ (Si–O–Si) and 550 cm^−1^ (Mg–O) from the aminoclay, suggesting the formation of an insulin-aminoclay complex [34]. EDX analysis of AC-Ins also indicated distinct components originating from insulin (S) and aminoclay (Si, Mg), confirming the integration of insulin and aminoclay into the nanoparticles (Fig. 1b). EDX mapping analysis examined the atomic distribution of Mg and S, distinct components of aminoclay and insulin, respectively. This also confirmed co-localization of insulin and aminoclay in AC-Ins nanoparticles (Fig. 1b). TEM was used to examine the morphology of nanoparticles. All nanoparticles exhibited spherical shapes and their size was comparable to that determined by dynamic light scattering (Fig. 1c).

Given that the structural stabilization of proteins is critical in the formulation development of protein drugs, the secondary structure of insulin entrapped in nanoparticles was examined by CD (circular dichroism) spectroscopy. As shown in Fig. 1d, the CD spectra and secondary structure contents of insulin released from nanoparticles were similar to those of native insulin, indicating that the conformational stability of insulin was maintained in the nanoparticles. Furthermore, these results were confirmed by deconvoluted amide I region of FT-IR spectra and the secondary structure contents of insulin released from nanoparticles (Additional file 1: Figure S2). This result suggests that the aminoclay-based core complex can serve as an effective support matrix to maintain the native structure of immobilized proteins [22, 24].

Collectively, aminoclay-based nanoparticles with/without surface coating were obtained with high entrapment efficiency (> 90%) in a spherical shape. All developed nanoparticles effectively maintained the conformational stability of the entrapped protein.

### In vitro drug release studies

**Fig. 2:**
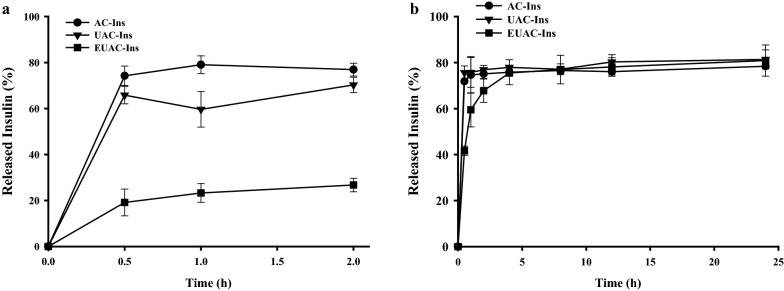
In vitro release profiles of insulin from different formulations at pH 1.2 (**a**) and at pH 6.8 (**b**) (mean ± SD, n = 3)

**Fig. 3:**
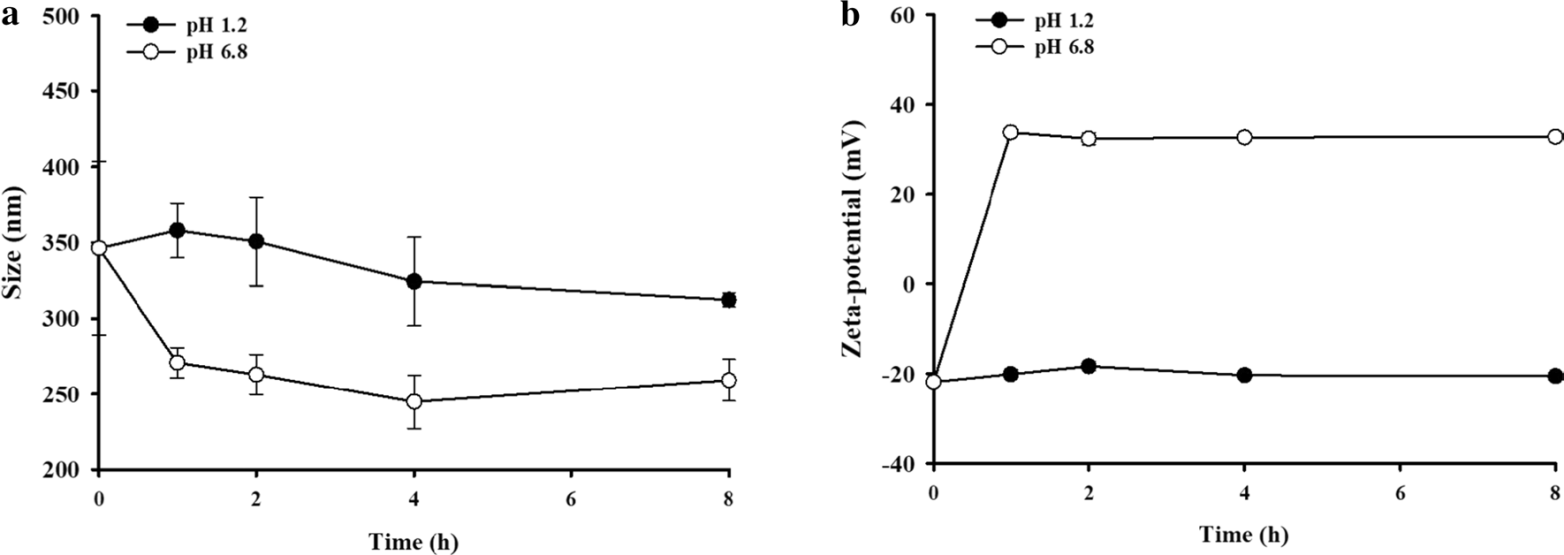
Variation in particle size and zeta potential of EUAC-Ins during incubation at different pHs (mean ± SD, n = 3)

In vitro drug release profiles of each nanoparticle were evaluated at pH 1.2 and 6.8 to reflect the gastric and small intestinal pH, respectively [35]. As shown in Fig. 2a, both AC-Ins and UAC-Ins exhibited rapid drug release at pH 1.2, where they released 65–74% of the drug within 30 min. In contrast, drug release from EUAC-Ins was suppressed up to 25% at pH 1.2 because the outer coating layer of Eudragit® L100 is insoluble at a pH of less than 6.0. Accordingly, drug release from EUAC-Ins increased up to 80% at pH 6.8 and was similar to the release from AC-Ins and UAC-Ins, probably due to dissolution of the outer coating layer at pH 6.8 (Fig. 2b). The removal of the Eudragit® L100 coating layer at pH 6.8 is also supported by the change in particle size and surface charge (Fig. 3). While the particle size of EUAC-Ins was maintained with minimal variation at pH 1.2, it rapidly decreased during the incubation at pH 6.8 as Eudragit® L100 is soluble at pH 6.8 (Fig. 3a). Consistently, the surface charge was reversed from negative to positive at pH 6.8 because the dissolution of Eudragit® L100 coating layer exposed the positively charged UEA-1 coating layer (Fig. 3b). The pH-dependent drug release characteristics of EUAC-Ins may prevent premature drug release in the harsh gastric environment and deliver more drugs into the small intestine.

### Protection against enzymatic degradation

**Fig. 4:**
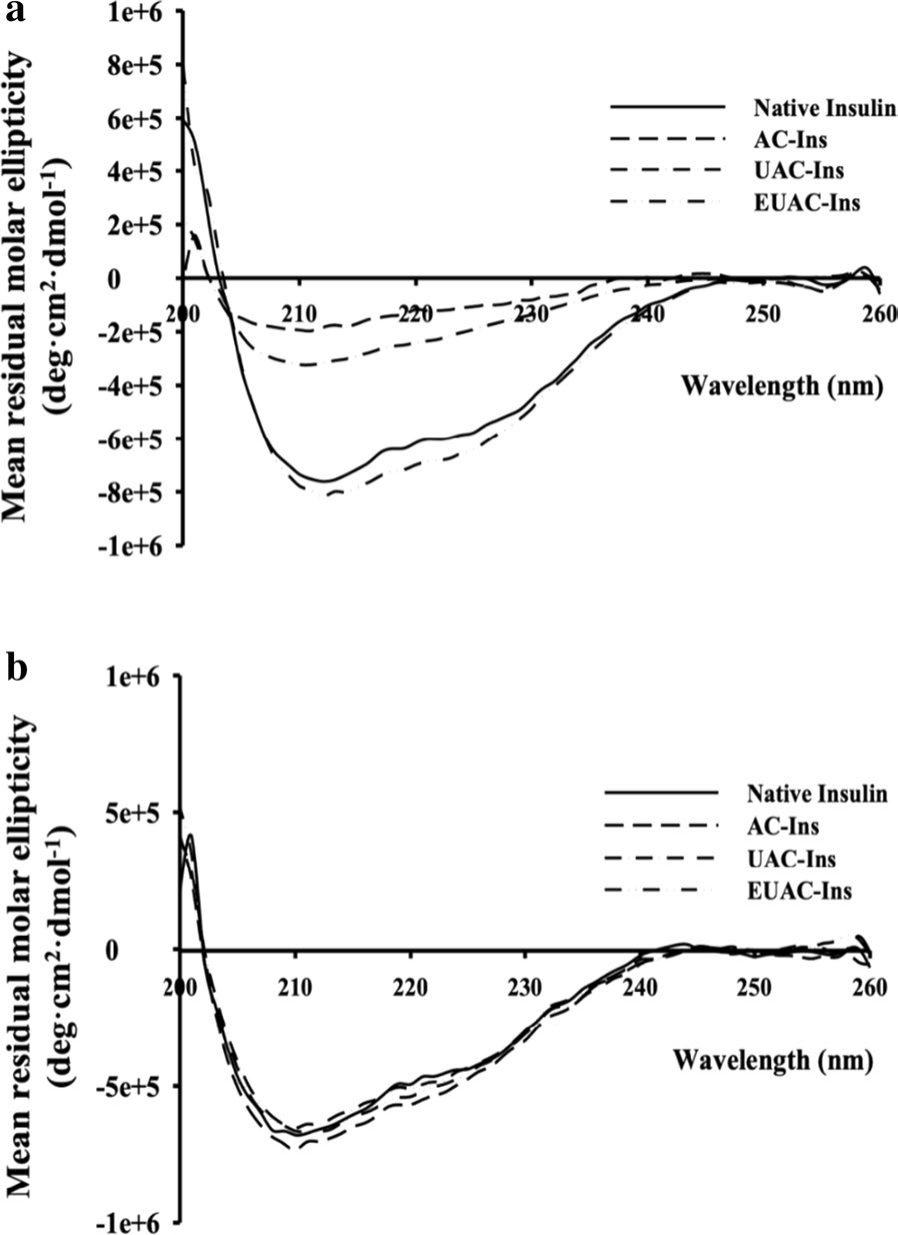
CD spectra of insulin released from different formulations after incubation in SGF for 1 h (**a**) and SIF for 2 h (**b**). At the end of incubation in SGF and SIF, each formulation was collected and incubated at pH 6.8. The released insulin underwent CD spectroscopic analysis

The protective effect of nanoparticles against gastrointestinal destabilization of insulin was evaluated in simulated gastrointestinal fluids containing proteolytic enzymes. The pH of simulated gastric fluids (SGF) and intestinal fluids (SIF) was adjusted to 1.2 and 7.4, respectively, to reflect the gastrointestinal pHs and maximize enzymatic activities, as the optimal pH for pepsin and trypsin is pH 1.0–2.0 and pH 7.0–8.0, respectively [35–37]. After incubating each formulation in SGF or SIF, the secondary structure of insulin entrapped in nanoparticles was examined by CD spectroscopy. Insulin entrapped in AC-Ins and UAC-Ins was completely destabilized in SGF mimicking conditions in the stomach, but it retained conformational stability in SIF (Fig. 4). In contrast, the secondary structure of insulin entrapped in EUAC-Ins was similar to that of native insulin in both SGF and SIF, suggesting that EUAC-Ins could protect entrapped insulin against enzymatic destabilization during the transition in the GI tract.

### Cellular transport studies

The effect of nanoparticles on the cellular uptake of insulin was examined in human intestinal epithelial cells (Caco-2 cells) and an in vitro human M cell model. Since Kernéis et al. [38] proposed the in vitro M cell-like model by co-culturing Caco-2 cells with lymphocytes isolated from murine PP’s, various cell culture models mimicking M cells have been proposed [38, 39]. Among them, the co-culture system of Caco-2 and Raji B cells is widely used as it is a reproducible and simplified model, based on entirely human cell lines, and does not require the isolation of primary cells or the use of animals [40]. This in vitro model exhibits the relevant characteristics of human M cells, including M cell-like morphology, particle transport, and expression of M cell-specific adhesion molecules [40]. Therefore, we used the co-culture system of Caco-2 cells and Raji B cells as an in vitro human M cell model. Before the transport studies, the effect of nanoparticles on cell viability was evaluated and none of the tested nanoparticles were cytotoxic at concentrations up to 1 mg/mL (Additional file 1: Figure S3).

**Fig. 5:**
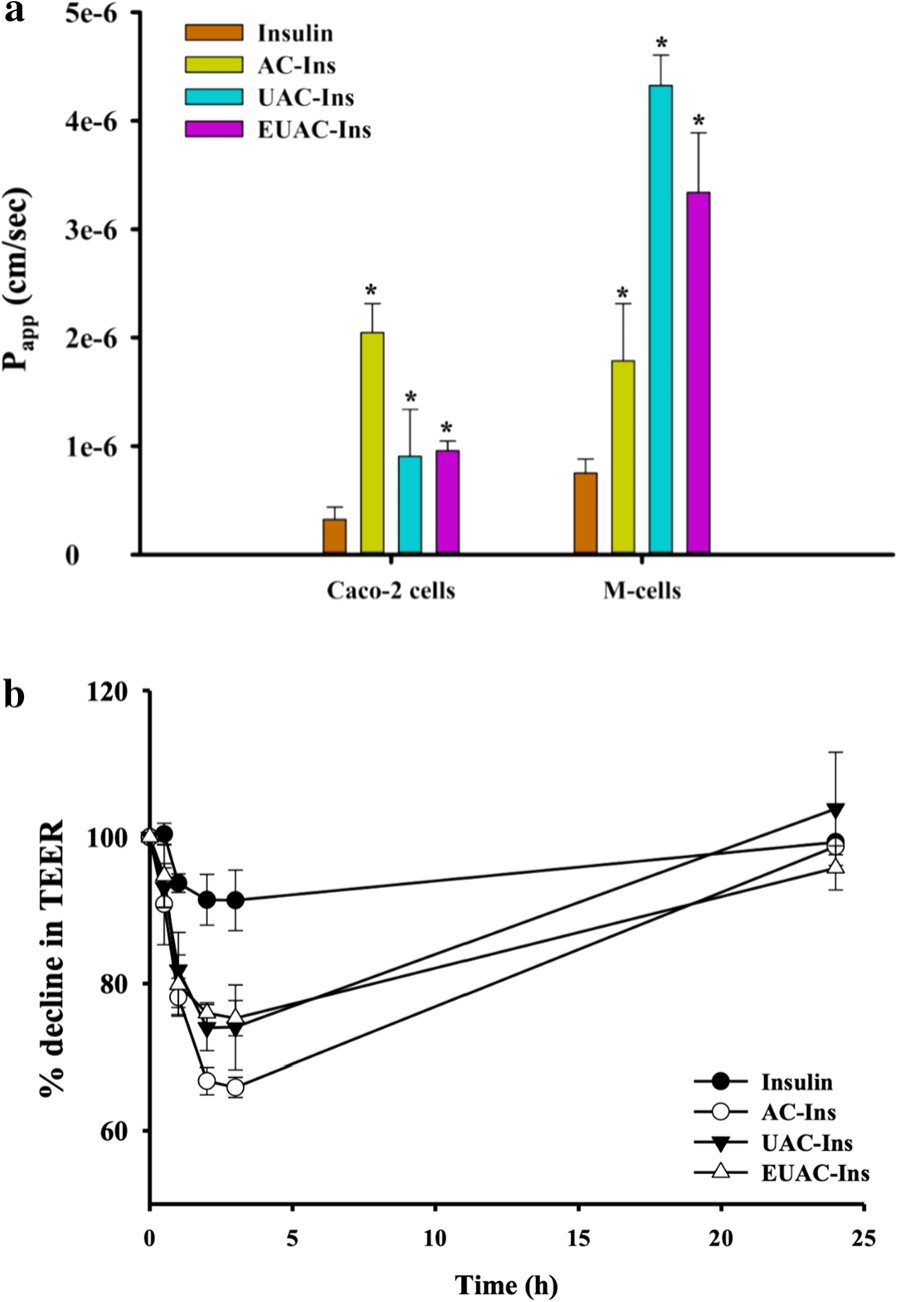
Drug transport studies in Caco-2 cells and M-cells (mean ± SD, n = 3). **a** Permeability of insulin from each formulation in Caco-2 cells and M cells. *p < 0.05 compared to free insulin solution; **b** the effect of nanoparticles on trans-epithelial electrical resistance (TEER) in Caco-2 cells. TEER values were measured during incubation in the presence and absence of nanoparticles. After 3 h of incubation, the drug solution was replaced by a fresh culture medium. The recovery of TEER values was monitored over time

The apparent permeability coefficients (P_app_) of insulin in different formulations were determined in Caco-2 cells and an in vitro human M cell model. As shown in Fig. 5a, all nanoparticles achieved a 2.8–6.3-fold higher drug permeability compared to free insulin in Caco-2 cells. This indicates that the developed nanoparticles may enhance drug transport across the intestinal epithelial membrane. As reported in our previous study [22], aminoclay likely has a reversible and transient effect on tight junction openings that enhances drug permeation. This study also indicates that TEER values significantly decrease in the presence of nanoparticles, but fully recover after nanoparticle removal at the end of the experiment (Fig. 5b). Among nanoparticles, AC-Ins exhibited the greatest effect on tight junction modulation and enhanced drug transport to the greatest extent in Caco-2 cells. In addition to the paracellular pathway, positively charged nanoparticles may interact with negatively charged membrane proteins to enhance nanoparticle-cell interactions.

Incorporation of UEA-1 into nanoparticles enhanced drug transport far more in in vitro human M cells than in Caco-2 cells. As shown in Fig. 5a, UEA-1-coated nanoparticles enhanced drug permeation to the greatest extent, facilitating drug transport approximately 4.4–5.8-fold in M cells as compared to free insulin. This result may be due to the high affinity of UEA-1 for differentiated M cells and the formation of a large “pocket” in the M cells [41–43].

These results suggest that the developed nanoparticles improve insulin transport across the intestinal epithelial membrane, particularly via M cells in intestinal PPs.

### Ex vivo intestinal absorption study in mice

**Fig. 6:**
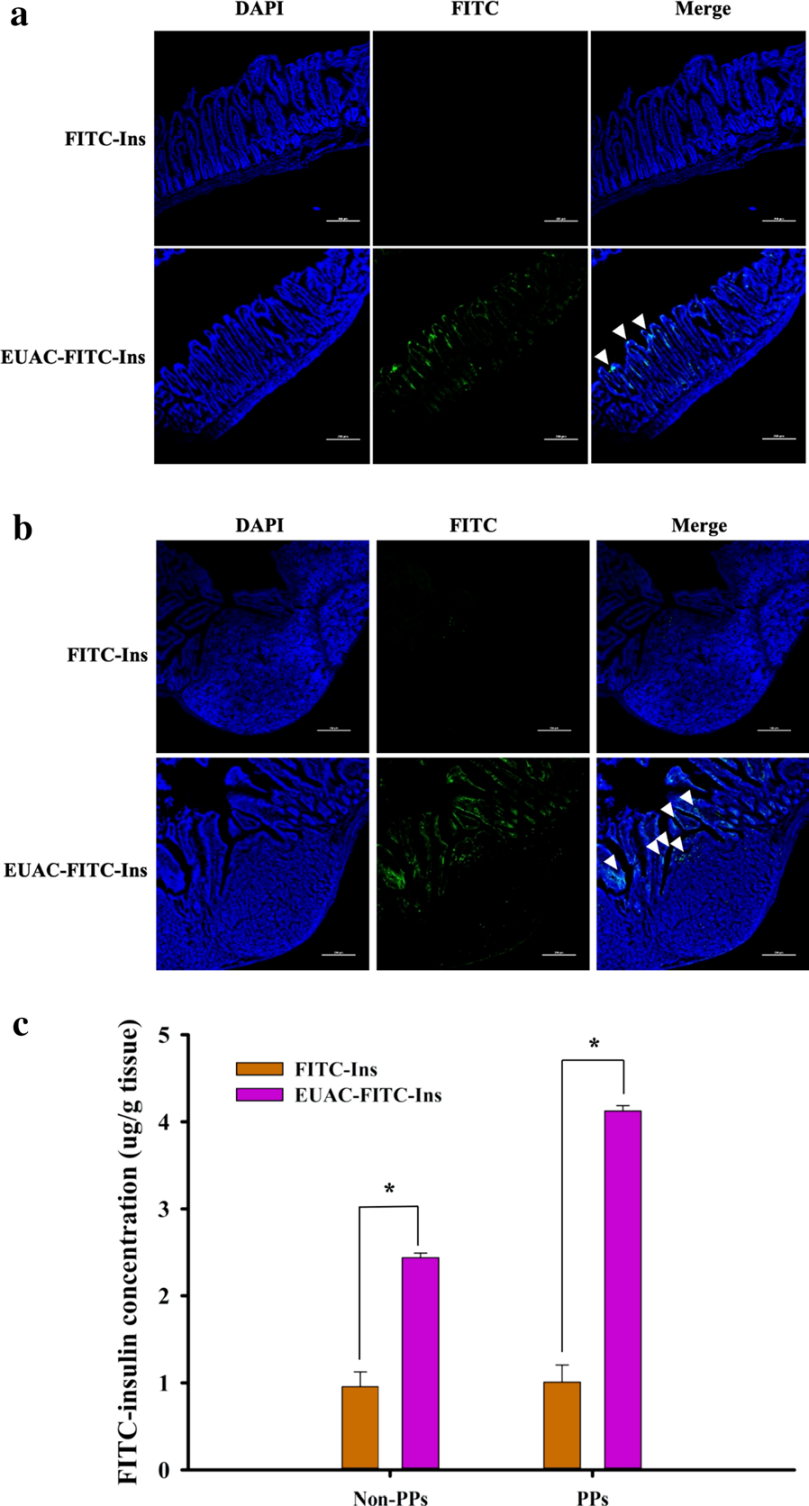
Intestinal localization of FITC-Insulin after oral administration of each formulation in mice. The dose was equivalent to 50 IU/kg FITC-Ins. **a**, **b** CLSM images of excised small intestinal epithelium with normal villi (non-PPs) (**a**) and intestinal Peyer’s patch (PPs) (**b**). White arrows indicate FITC-labeled insulin and the scale bar represents 200 μm. **c** Quantitation of FITC-Insulin in intestinal tissues (mean ± SD, n = 3). *p < 0.05

The effectiveness of nanoparticles as an oral insulin delivery system was examined by an ex-vivo intestinal drug absorption study in mice. After oral administration of FITC-insulin loaded-nanoparticles (EUAC-FITC-Ins) or free insulin solution (FITC-Ins), gastrointestinal drug absorption was examined in small intestinal epithelium with normal villi (non-PPs) and PPs. As illustrated in Fig. 6, fluorescence signals were negligible in both non-PPs and PPs after oral administration of free FITC-Ins solution, which may be attributed to the instability of insulin in the GI tract and low drug permeability. In contrast, EUAC-FITC-Ins achieved significantly higher intestinal absorption, particularly in intestinal PPs (Fig. 6).

This result may be explained by several factors. First, a pH-dependent polymer coating may protect entrapped insulin against acidic and enzymatic destabilization in the stomach. Second, after the dissolution of the Eudragit® L-100 coating layer in the small intestine, exposed UEA-1 on the nanoparticle surface might interact with M cells in the PPs of the intestine and facilitate drug transport. In addition, the modulation of tight junctions by aminoclay may contribute to enhanced drug absorption across the intestinal epithelium.

In conjunction with an in vitro drug transport study, these results suggest that EUAC-Ins improves the oral delivery of insulin.

### Hypoglycemic effect in diabetic mice

The hypoglycemic effect of orally-administered nanoparticles was evaluated and compared to that of free insulin in STZ-induced diabetic mice. After SC injection of insulin solution, blood glucose concentrations rapidly reduced to 20% of the initial blood glucose levels within 1 h and then gradually recovered to the initial blood glucose level within 8 h. In contrast, an orally administered insulin solution did not exhibit any hypoglycemic effect, probably due to instability in the GI tract and low membrane permeability.

**Fig. 7:**
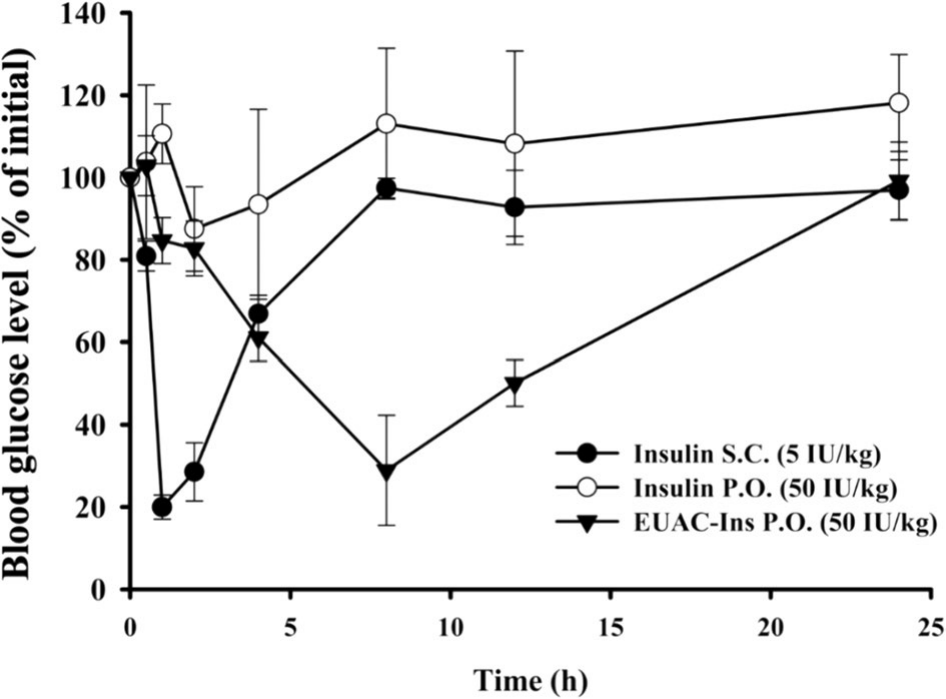
Hypoglycemic effect following oral or subcutaneous injection of each formulation in diabetic mice (mean ± SD, n = 3)

In parallel to the in vitro and ex vivo studies described above, the improved GI stability and permeability of insulin via EUAC-Ins nanoparticles significantly enhanced the hypoglycemic effect of orally administered insulin in STZ-induced diabetic mice. As shown in Fig. 7, oral administration of EUAC-Ins significantly decreased the blood glucose concentration to 30% of the initial blood glucose level within 8 h, and then gradually recovered to the initial blood glucose concentrations within 24 h. Compared to SC injection of insulin solution, EUAC-Ins lowered blood glucose concentration more slowly but achieved the hypoglycemic effect for a longer time. This may be because of delayed drug release from EUAC-Ins until the pH-dependent outer coating layer dissolves in the small intestine. The results suggest that EUAC-Ins is an effective oral insulin delivery system.

Previous studies also reported the hypoglycemic effect of oral insulin formulations in diabetes models, demonstrating that they effectively lowered the blood glucose levels to 30–76% of the initial blood glucose level [43–48]. Woitiski et al. [48] developed multilayered nanoparticles for oral insulin delivery and demonstrated that nanoparticulated insulin reduced plasma glucose levels to 40% of the basal value within 12 h after oral administration. Jin et al. [49] developed goblet cell-targeting nanoparticles for the oral absorption of insulin by using trimethyl-chitosan chloride modified with a CSKSSDYQC targeting peptide. These nanoparticles exhibited good targeting efficiency to goblet cells, with enhanced drug permeation, but showed a relatively weak hypoglycemic effect with the maximal blood glucose depression of 28% within 3 h after oral administration [49]. Although it is difficult to make a direct comparison between our results and the literature, EUAC-Ins in the present study achieved a strong hypoglycemic effect comparable to SC injection over a longer period and is a promising oral insulin delivery system.

## Conclusion

In the present study, EUAC-Ins was developed as an M cell-targeted nanocomposite system for effective oral insulin delivery via dual coating of an aminoclay-insulin core complex with UEA-1 and Eudragit® L-100. EUAC-Ins was obtained as a nano-sized particle with high entrapment efficiency (> 90%). EUAC-Ins improved the GI stability of insulin and drug transport across the intestinal membrane, leading to enhanced intestinal drug absorption, particularly via M cells in intestinal PPs. Consequently, oral administration of EUAC-Ins exhibited a significant hypoglycemic effect in the diabetic mouse model, while oral administration of free insulin did not show efficacy. These results suggest that EUAC-Ins is a promising oral insulin delivery system.

## Methods

### Materials

Human recombinant insulin was purchased from FUJIFILM Wako Pure Chemical Co. (Osaka, Japan). Eudragit® L100 was obtained from Evonik Korea Ltd. (Seoul, Korea). FITC-labeled Insulin, pepsin, trypsin, 3-aminopropyltriethoxysilane (APTES, 99%), and 4-kDa FITC-conjugated dextran (FD4) were purchased from Sigma-Aldrich Co. (St Louis, MO, USA). Dulbecco’s Modified Eagle’s medium (DMEM), Roswell Park Memorial Institute (RPMI)-1640 medium, Hank’s balanced salt solution (HBSS), non-essential amino acids, fetal bovine serum (FBS), penicillin–streptomycin, and all other reagents used in the cell culture studies were obtained from GE Healthcare Life Sciences (South Logan, UT, USA). 4ʹ,6-Diamidino-2-phenylindole (DAPI) was purchased from Invitrogen Molecular Probes (Karlsruhe, Germany). Paraformaldehyde was purchased from Merck KGaA (Darmstadt, Germany). Magnesium chloride hexahydrate (98%) and other inorganic salts were purchased from Junsei Chemical Co., Ltd (Tokyo, Japan). All other chemicals and reagents were HPLC-grade.

Caco-2 cells (human epithelial colorectal adenocarcinoma cells) were purchased from the Korean Cell Line Bank (Seoul, Korea). Raji cells were obtained from American Type Culture Collection (ATCC, USA). Caco-2 cells were grown in DMEM containing 10% FBS, 1% nonessential amino acid, and 1% antibiotics. Raji cells were grown in RPMI-1640 containing 10% FBS with 1% antibiotics. Cells were incubated at 37 °C in an atmosphere of 5% CO_2_ and 90% relative humidity.

### Preparation of nanoparticles

3-Aminopropyl-functionalized magnesium phyllosilicate (aminoclay) was synthesized as previously described [23]. In brief, magnesium chloride (1.68 g) was dissolved in ethanol (40 g). 3-Aminopropyltriethoxysilane (2.6 mL) was then added dropwise under vigorous stirring at 250 rpm to result in the rapid formation of a white precipitate. After stirring overnight, the precipitate was separated by centrifugation, washed three times with ethanol, and dried at 40 °C.

Aminoclay was dispersed in water and exfoliated by ultrasonication for 10 min. Insulin (10 mg/mL) was dissolved in 10 mM HCl and then diluted with 100 mM Tris–HCl (pH 8.0) to obtain a final concentration of 5 mg/mL. This insulin solution was added drop-wise to an aqueous suspension of aminoclay (10 mg/mL) at a drug/clay ratio of 1:3 while stirring at 300 rpm. After three hours of stirring, the white precipitate was left to age for an hour, separated by centrifugation (22,250×*g*) for 10 min, and dried at room temperature under vacuum. The obtained AC-Ins (8 mg/mL) was suspended in 2.5 mM HCl and added dropwise into an equal volume of UEA-1 solution (2 mg/mL), forming a UEA-1 coated AC-Ins complex (UAC-Ins). After stirring at 300 rpm for 30 min, the resultant nanoparticles (UAC-Ins) were collected by centrifugation at 77,100×*g* for 15 min. Finally, UAC-Ins was re-dispersed in 5 mM HCl and added into an equal volume of Eudragit® L-100 in ethanol while stirring at 300 rpm for 30 min. After ultra-centrifugation (77,100×*g*) for 15 min, the collected nanoparticles (EUAC-Ins) were dried at room temperature under vacuum.

For the bioimaging study, FITC-insulin-loaded nanoparticles were prepared following the procedure described above.

### Structural characterization of nanoparticles

The particle size, zeta potential, and polydispersity index (PDI) of the nanoparticles were determined using a Zetasizer Nano-ZS90 (Malvern Instruments, Malvern, UK). Zeta potential was measured at pH 3.0. The entrapment efficiency (EE) of the nanoparticles was calculated as follows:

$${\text{EE}} \left( \% \right) = \frac{{{\text{Drug amount initially added}} - {\text{Drug amount in supernatant}}}}{{{\text{Drug amount initially added}}}} \times 100.$$

The structural characteristics of nanoparticles were examined by FT-IR with a ZnSe crystal accessory (Nicolet™ iS™ 5; Thermo Fisher Scientific, Waltham, MA, USA). The FT-IR spectrum of each sample was obtained over a wavenumber range of 4000–500 cm^−1^ with 64 scans at a resolution of 4 cm^−1^. Circular dichroism (CD) analysis (Chirascan™-Plus Spectrometer; Applied Photophysics, Surrey, UK) was used to examine the secondary structure of insulin released from the nanoparticles. Wavelength spectra were collected from 200 to 260 nm at 25 °C with a bandwidth of 1 nm and a light path length of 0.5 mm. The CD spectra from each sample were deconvoluted and the secondary structure content was quantitated using CDNN 2.1 software [50].

The morphologic characteristics of nanoparticles were monitored by transmission electron microscopy (TEM) (JEM-F200; JEOL Ltd., Tokyo, Japan). The compositional elements of nanoparticles were examined by an energy dispersive X-ray spectrometer (EDX). TEM and EDX analysis were performed at the National Center for Inter-University Research Facilities (NCIRF) at Seoul National University (Seoul, Korea).

### In vitro drug release studies

The drug release profiles of nanoparticles were evaluated at pH 1.2 and 6.8. Nanoparticles (equivalent to 0.25 mg/mL of insulin) were incubated in a release medium at 37 °C while stirring at 100 rpm. At predetermined time points, samples were collected and centrifuged at 22,250×*g* for 10 min. Supernatants were analyzed by HPLC to determine the amount of drug released.

The in vitro stability of EUAC-Ins was examined during incubation at pH 1.2 and 6.8. EUAC-Ins (5 mg) was added to each buffer system (10 mL) at different pHs and incubated at 37 °C. At predetermined time points, samples were collected and the particle size and zeta potential were measured by using a Zetasizer Nano-ZS90 (Malvern Instruments, Malvern, UK).

### Protection against enzymatic degradation

The structural stability of insulin in the nanoparticles was examined in simulated gastric juice (SGF, pH 1.2 with 5 µg/mL pepsin) and intestinal fluid (SIF, pH 7.4 with 50 µg/mL trypsin). After incubating the nanoparticles in SGF or SIF at 37 °C for the designated time, the enzymatic reaction was terminated by adding either 0.2 mL of 0.2 M NaOH into SGF or 0.1 M HCl into SIF. Nanoparticles were collected by centrifugation (22,250×*g*) for 10 min, and insulin in the nanoparticles was extracted in a phosphate buffer (pH 7.4) for 2 h. The secondary structure of the insulin extracted from nanoparticles was analyzed by CD spectroscopy.

### Transport studies

An in vitro human M cell model was established by preparing a co-culture of Caco-2 and Raji cells, as reported by Kim et al. with slight modifications [42]. Briefly, Caco-2 cells were seeded at a density of 5.0 × 10^5^ cells/well in 12-well plates (with an insert surface area of 1.12 cm^2^). After 14 days of mono-culturing Caco-2 cells, Raji cells suspended in RPMI/DMEM (1:2) mixture were added to the basolateral side of the inserts at a density of 5 × 10^5^ cells/well. On day 21 after seeding, both co-cultures and monocultures were used for experiments. The integrity of the cell monolayers was monitored by measuring trans-epithelial electrical resistance (TEER) using an epithelial tissue voltohmmeter (MillicellERS-2, MerckKGaA, Darmstadt, Germany).

Before the transport studies, inserts were washed twice with HBSS. The HBSS in the apical side was replaced by a drug solution containing each formulation (equivalent to 10 µg/mL of insulin). At each time point, 100 µL of the samples were collected from the basolateral compartment and replenished by an equal volume of fresh HBSS to maintain a constant volume. Drug concentrations were determined by LC–MS/MS. The apparent permeability coefficient (Papp) was calculated using the following equation:

$${\text{P}}_{{{\text{app}}}} = {\text{dQ}}/{\text{dt}} \times 1/{\text{AC}}_{0} ,$$

where dQ/dt is the cumulative drug amount in the basolateral compartment as a function of time, A is the surface area of the membrane filter, and C_0_ is the initial drug concentration. TEER values were measured during and after the experiment. At the end of the experiment, the drug solution was removed, both apical and basolateral compartments were replenished by fresh HBSS, and TEER values were monitored for up to 24 h.

### Ex vivo intestinal absorption study in mice

Animal studies were conducted in accordance with the “Guiding Principles in the Use of Animals in Toxicology,” adopted by the Society of Toxicology (USA), and the study protocol was approved by the review committee of Dongguk University (IACUC-2017-016-4). C57BL/6J (25 ± 2 g) were obtained from Orient Bio Inc. (Seongnam, Korea). Mice were fasted for 12 h before the experiment but were given free access to tap water. An aqueous suspension of each formulation was administered to the mice by oral gavage at a dose equivalent to 50 IU/kg FITC-Ins. Mice were sacrificed 3 h post-dose, and the small intestine was excised immediately after laparotomy. Peyer’s patches (PPs) were also isolated from the sub-epithelium of the small intestine, as described in a previous study [51]. The obtained intestinal segments were washed with PBS to remove the luminal content. Each tissue was fixed with 4% paraformaldehyde overnight. Fixed tissues were placed in a 30% sucrose solution, and each tissue was cryo-sectioned followed by confocal microscopic examination (Nikon C1, Nikon, Tokyo, Japan) to visualize the localization of the fluorescence-labeled insulin.

For quantitative analysis of the drug amount, each tissue was homogenized in PBS and then mixed with twice the volume of methanol. After vigorous stirring for 2 min, the mixture was centrifuged at 16,600×*g* for 10 min. The supernatant was collected and dried under vacuum. The dried residue was reconstituted with PBS and then quantified by a fluorescence plate reader (SpectraMax M2, Molecular Devices, LCC., San Jose, CA).

### Hypoglycemic effect in diabetic mice

The efficacy of orally administered nanoparticles or free insulin was evaluated in streptozotocin (STZ)-induced mice and compared to a subcutaneously injected insulin solution. Male C57BL/6 (25 ± 3 g) mice were purchased from Orient Bio Inc. (Seongnam, Korea). The experimental protocol was approved by the review committee of Dongguk University (IACUC-2017-016-3). Mice were fasted for 4 h before STZ injection. As previously reported [52], diabetes was induced by an intraperitoneal injection of STZ (in 50 mM citrate buffer solution, pH 4.5) at a dose of 200 mg/kg. After 10 days, blood was collected from the saphenous vein to measure blood glucose levels using a Roche glucose meter (ACCU-CHEK® guide). Mice with fasting blood glucose over 500 mg/dL were used as diabetic mouse models [52].

All mice were fasted for 6 h before the experiment but were freely allowed water. STZ-induced diabetic mice were randomly divided into three groups. Group 1 was administered an insulin solution at a dose of 5 IU/kg via subcutaneous (SC) injection. Groups 2 and 3 were given orally insulin solution or EUAC-Ins, respectively, at a dose equivalent to 50 IU/kg of insulin. Blood samples were collected from the saphenous vein at each time point. Blood glucose levels were measured using a Roche glucose meter (ACCU-CHEK® guide) and were expressed as percentages of the initial glucose levels.

### Analytical assay

Drug concentrations were analyzed by HPLC and LC–MS/MS. The HPLC system (Flexar; Perkin Elmer, MA, USA) consisted of an automatic injector, a UV detector, and two solvent delivery pumps. Samples were injected into the HPLC system connected to a column (Gemini C18, 4.6 × 150 mm, 5 μm; Phenomenex, Torrance, CA, USA). Chromatographic separation was achieved by eluting the mobile phase (acetonitrile: 0.1% trifluoroacetic acid = 30:70, v/v) at a flow rate of 1 mL/min, with a column temperature of 30 °C. Prednisone was used as an internal standard. The detection wavelength was set to 215 nm. The calibration curve was obtained in the range of 5–200 µg/mL with good linearity (R^2^ > 0.999).

For LC–MS/MS analysis, chromatographic separation was achieved with a C18 column (4.6 × 150 mm, 5 μm; Phenomenex, CA, USA) at 40 °C by eluting a mobile phase (0.1% formic acid in acetonitrile:0.1% formic acid in water = 60:40, v/v) at a flow rate of 1 mL/min. Mass spectrometric detection was achieved using positive ion electrospray ionization in the multiple-reaction monitoring (MRM) mode with an ABSciex API 4000 triple quadrupole mass spectrometer (ABSciex, Framingham, MA, USA). The precursor/product ion pair (m/z) was 1162.6/143.2 for insulin and 271.2/155.1 for the internal standard (tolbutamide). The calibration curve was obtained in the range of 20–1000 ng/mL with good linearity (R^2^ > 9996).

### Statistical analysis

All data are expressed as mean ± standard deviation (SD). Statistical analyses were performed using one-way ANOVA followed by Dunnett’s test. Values of p < 0.05 were considered significantly different.

## Supplementary Information

**Additional file 1: Figure S1.** Confocal microscopy images of AC-Ins (A) and AC-Ins coated with FITC-labeled UEA-1 (B). Green signals represent the FITC-labeled UEA-1. Scale bar represents 2.5 µm. **Figure S2.** Deconvoluted amide I region of FT-IR spectra (A) and the secondary structure contents (B) of insulin released from different formulations after incubation at pH 6.8 for 8 h. Deconvolution of the amide I region of the spectra was performed by using Omnic software ver.1.08 (Thermo Fisher Scientific, Waltham, MA, USA). Secondary structure contents were assigned from deconvolution peak positions as alpha-helix at 1656 cm^−1^, random at 1647 cm^−1^, beta-sheet at 1630 cm^−1^, and beta-turn at 1677 cm^−1^. **Figure S3.** Effect of aminoclay and each formulation on cell viability in Caco-2 cells. Cytotoxic effects were determined after 48 h of incubation (mean ± SD, n = 3).

## Data Availability

All data generated or analysed during this study are included in this published article.

## Abbreviations

GI: Gastrointestinal
UEA-1: *Ulex europaeus agglutinin* 1
FT-IR: Fourier transform-infrared spectroscopy
EDX: Energy dispersive X-ray spectroscopy
CD: Circular dichroism
SGF: Simulated gastric fluid
SIF: Simulated intestinal fluid
P_app_: Apparent permeability coefficients
PPs: Peyer’s patches
