# Supplementary material for: Development of an M cell targeted nanocomposite system for effective oral protein delivery: preparation, in vitro and in vivo characterization
Source: J Nanobiotechnology. 2021 Jan 9;19:15. doi: 10.1186/s12951-020-00750-y (PMC7796596; doi:10.1186/s12951-020-00750-y)
Supplement: Supplementary file 1 — Additional file 1: Figure S1. Confocal microscopy images of AC-Ins (A) and AC-Ins coated with FITC-labeled UEA-1 (B). Green signals represent the FITC-labeled UEA-1. Scale bar represents 2.5 µm. Figure S2. Deconvoluted amide I region of FT-IR spectra (A) and the secondary structure contents (B) of insulin released from different formulations after incubation at pH 6.8 for 8 h. Deconvolution of the amide I region of the spectra was performed by using Omnic software ver.1.08 (Thermo Fisher Scientific, Waltham, MA, USA). Secondary structure contents were assigned from deconvolution peak positions as alpha-helix at 1656 cm−1, random at 1647 cm−1, beta-sheet at 1630 cm−1, and beta-turn at 1677 cm−1. Figure S3. Effect of aminoclay and each formulation on cell viability in Caco-2 cells. Cytotoxic effects were determined after 48 h of incubation (mean ± SD, n = 3). [file 12951_2020_750_MOESM1_ESM.pdf]

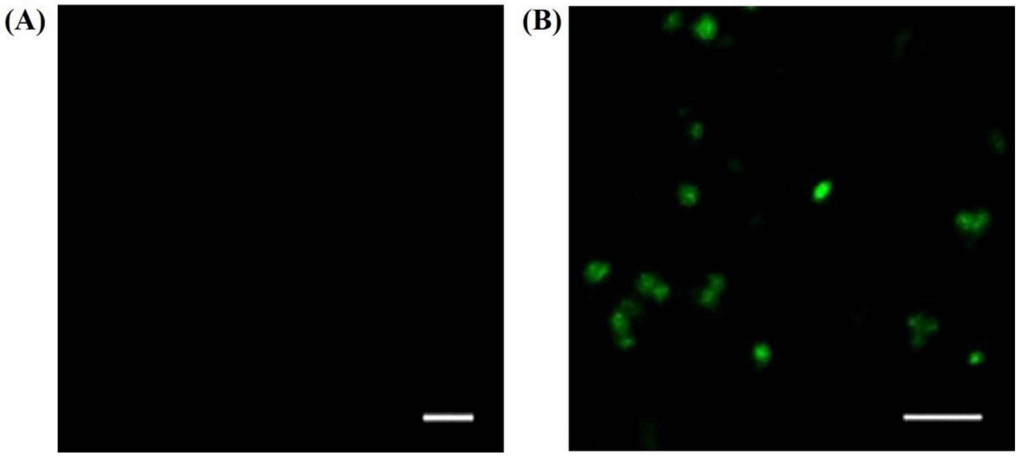

Figure S1. Confocal microscopy images of AC-Ins (A) and AC-Ins coated with FITC-labeled UEA-1 (B). Green signals represent the FITC-labeled UEA-1. Scale bar represents 2.5 μm.

(A)

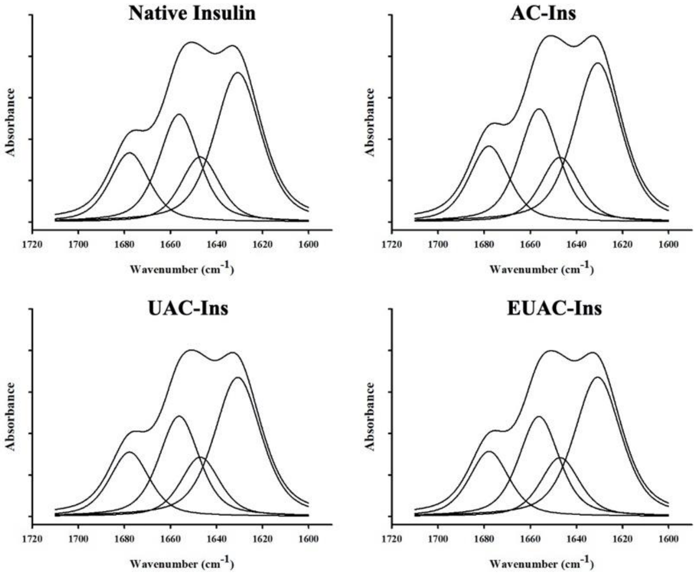

(B)

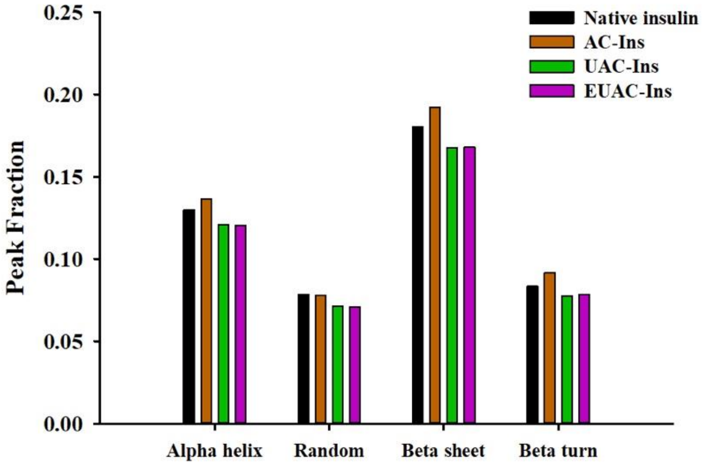

Figure S2. Deconvoluted amide I region of FT-IR spectra (A) and the secondary structure contents (B) of insulin released from different formulations after incubation at pH 6.8 for 8 h. Deconvolution of the amide I region of the spectra was performed by using Omnic software ver.1.08 (Thermo Fisher Scientific, Waltham, MA, USA). Secondary structure contents were assigned from deconvolution peak positions as alpha-helix at 1656cm^{-1}, random at 1647cm^{-1}, beta-sheet at 1630cm^{-1}, and beta-turn at 1677cm^{-1}.

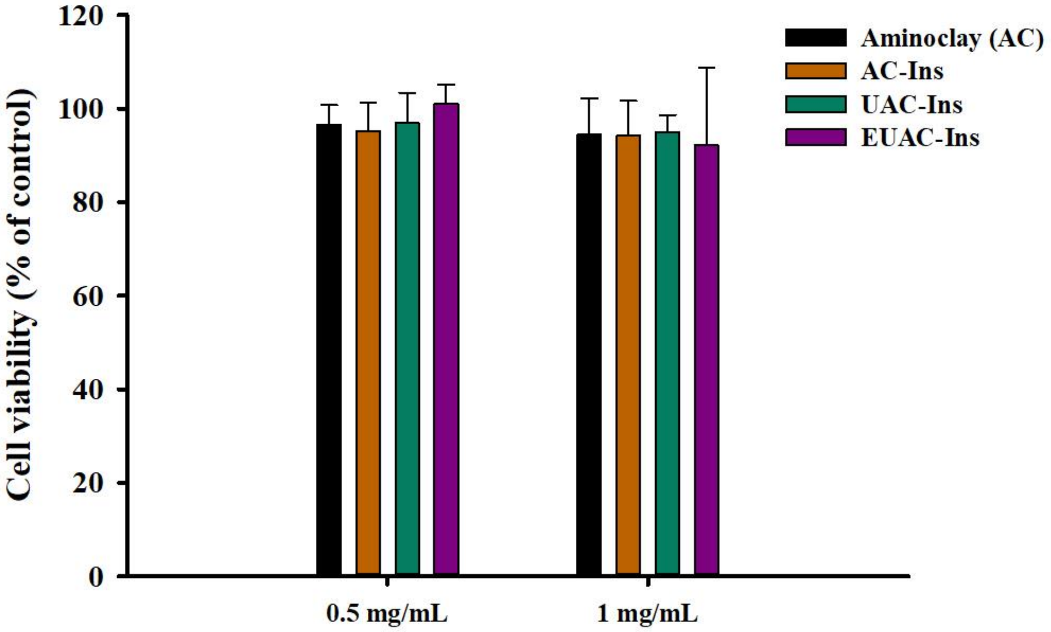

Figure S3. Effect of aminoclay and each formulation on cell viability in Caco-2 cells. Cytotoxic effects were determined after 48 h of incubation (mean ± SD, n = 3).
